# Hearing Loss in a Mouse Model of 22q11.2 Deletion Syndrome

**DOI:** 10.1371/journal.pone.0080104

**Published:** 2013-11-14

**Authors:** Jennifer C. Fuchs, Fhatarah A. Zinnamon, Ruth R. Taylor, Sarah Ivins, Peter J. Scambler, Andrew Forge, Abigail S. Tucker, Jennifer F. Linden

**Affiliations:** 1 Craniofacial Development & Stem Cell Biology, King's College London, London, United Kingdom; 2 Ear Institute, University College London, London, United Kingdom; 3 Institute of Child Health, University College London, London, United Kingdom; 4 Department of Neuroscience, Physiology & Pharmacology, University College London, London, United Kingdom; IGBMC/ICS, France

## Abstract

22q11.2 Deletion Syndrome (22q11DS) arises from an interstitial chromosomal microdeletion encompassing at least 30 genes. This disorder is one of the most significant known cytogenetic risk factors for schizophrenia, and can also cause heart abnormalities, cognitive deficits, hearing difficulties, and a variety of other medical problems. The *Df1/+* hemizygous knockout mouse, a model for human 22q11DS, recapitulates many of the deficits observed in the human syndrome including heart defects, impaired memory, and abnormal auditory sensorimotor gating. Here we show that *Df1/+* mice, like human 22q11DS patients, have substantial rates of hearing loss arising from chronic middle ear infection. Auditory brainstem response (ABR) measurements revealed significant elevation of click-response thresholds in 48% of *Df1/+* mice, often in only one ear. Anatomical and histological analysis of the middle ear demonstrated no gross structural abnormalities, but frequent signs of otitis media (OM, chronic inflammation of the middle ear), including excessive effusion and thickened mucosa. In mice for which both *in vivo* ABR thresholds and *post mortem* middle-ear histology were obtained, the severity of signs of OM correlated directly with the level of hearing impairment. These results suggest that abnormal auditory sensorimotor gating previously reported in mouse models of 22q11DS could arise from abnormalities in auditory processing. Furthermore, the findings indicate that *Df1/+* mice are an excellent model for increased risk of OM in human 22q11DS patients. Given the frequently monaural nature of OM in *Df1/+* mice, these animals could also be a powerful tool for investigating the interplay between genetic and environmental causes of OM.

## Introduction

22q11.2 Deletion Syndrome (22q11DS, OMIM #188400), also commonly known as DiGeorge Syndrome or Velo-Cardio-Facial Syndrome, is a genetic disorder that results from an approximately 1.5-3Mb congenital multigene deletion on the long arm of chromosome 22, which includes the gene for T-Box Transcription factor 1 (*TBX1*) [1,2]. 22q11DS occurs in 1:4000 live births, making it the most common interstitial deletion syndrome and the second most common chromosomal abnormality after Down's Syndrome [3]. Typical physical findings in 22q11DS patients include defects in cardiovascular [4], thymic, parathyroid and craniofacial [5] structures derived from the pharyngeal arches and pouches [6]. In addition, 22q11DS is associated with high frequencies (80–100%) of neurocognitive disabilities [7], and it is one of few cytogenetic abnormalities that occurs in tandem with a psychiatric disease [3]. The syndrome is one of the highest known risk factors for schizophrenia, as 25-30% of 22q11DS patients develop schizophrenia during adolescence or adulthood [8]. 

22q11DS is also a risk factor for development of otitis media (OM) [9]. OM is inflammation of the middle ear cavity (MEC), often presenting with pain and fever. It is the most common disease in young children worldwide, occurring at least once before the age of two in 90% of infants in the developed world [10]. OM is typically classified as either acute or chronic. Acute otitis media is associated with a bacterial infection and often resolves spontaneously within three months. However, in some cases acute OM is followed by otitis media with effusion (OME) that can become chronic [11]. OME is characterized by excessive effusion that accumulates in the MEC, and the absence of obvious signs of acute infection. Excessive effusion often leads to conductive hearing loss, which in severe cases can become permanent due to erosion of the middle ear ossicles [12] . Even in less severe cases, conductive hearing loss due to OME can interfere with speech and language development. The usual treatment and also the most common operation in the United Kingdom is the insertion of grommets into the tympanic membrane to permit ventilation and drainage of exudates from the MEC [13]. Risk factors for OM include infection, altered immune status, exposure to tobacco smoke, and anatomical defects such as cleft palate [14] . In addition, although the pathogenesis of OM is multifactorial, a role for genetic predisposition is increasingly recognized [15,16]. In 22q11DS, studies have shown that a majority of patients have a history of chronic or recurrent OM [17,18].

The hemizygous Df1-knockout mouse (*Df1/+*) was genetically engineered to be a model for human 22q11DS; it carries a multigene deletion in a region of mouse chromosome 16 that is orthologous to the 22q11.2 region in humans [19,20]. Although the region is highly conserved, several ancestral rearrangements have led to changes in gene order, and so the deletion in *Df1/+* mice encompasses 18 of the protein-encoding genes deleted in human 22q11DS. *Df1/+* mice have proven to be an excellent model for major developmental defects in human 22q11DS such as cardiovascular abnormalities [21] and thymic or parathyroid defects [22], although no gross craniofacial abnormalities such as cleft palate have been reported. Furthermore, both *Df1/+* mice and other mouse models of 22q11DS have been found to show cognitive and behavioural abnormalities associated with human 22q11DS and schizophrenia, including reduced auditory sensorimotor gating [23-25]. Modern tests of sensorimotor gating depend on the ability to hear, and previous studies have presented some evidence for normal hearing in *Df1/+* mice and similar mouse models [23-25]. However, mice heterozygous for *Tbx1*, one of the genes involved in the multigene deletion and the most likely candidate gene responsible for the pharyngeal arch-derived defects in 22q11DS, have been shown to suffer frequent middle ear inflammation with associated conductive hearing loss [26]. 

Here, we aimed to resolve this discrepancy in the literature, using auditory brainstem response (ABR) measurement to assess hearing capability in adult *Df1/+* mice and their WT littermates. To obtain data from a large population of age-matched *Df1/+* and WT mice, we focused on measurement of click-evoked ABR thresholds, a simple and rapid electroencephalographic measure of peripheral and early central auditory activity that could be obtained *in vivo* from each ear for all animals in a litter in a single day. We found that click-evoked ABR thresholds were significantly elevated in 48% of the *Df1/+* animals, often in only one ear. Anatomical and histological analysis of the middle ear revealed a high incidence of OME in *Df1/+* mice, which correlated directly with elevated ABR thresholds. We conclude that *Df1/+* mice, like human 22q11DS patients, are susceptible to otitis media and conductive hearing loss. These results suggest that studies of abnormal auditory sensorimotor gating in *Df1/+* mice need to be revisited using more sensitive assays for hearing loss, and also that *Df1/+* mice are a potentially powerful animal model for studying the genetic and environmental causes of otitis media.

## Results

### Elevated ABR thresholds in both male and female *Df1/*+ mice

The auditory brainstem response is an electroencephalographic signal arising from sound-evoked activity in neuronal circuits of the ascending central auditory pathway. ABRs evoked by click stimuli were recorded in 44 *Df1/+* mice (24 male, 20 female) and 43 WT littermates (24 male, 19 female), ranging in age from 8 to 40 weeks old. Measurements were taken once in each animal in either one or both ears, under free-field conditions with an ear plug in the opposite ear. Both left and right ears were tested in 31 of the *Df1/+* and 23 of the WT animals, and one ear only in 13 *Df1/+* and 20 WT mice. The ABR database therefore consisted of a total of 75 *Df1/+* and 66 WT ABR recordings. Click ABR thresholds were determined for each recording, and judged to be the lowest click intensity at which characteristic peaks of the ABR waveform could be observed (Figure S1).

**Figure 1:**
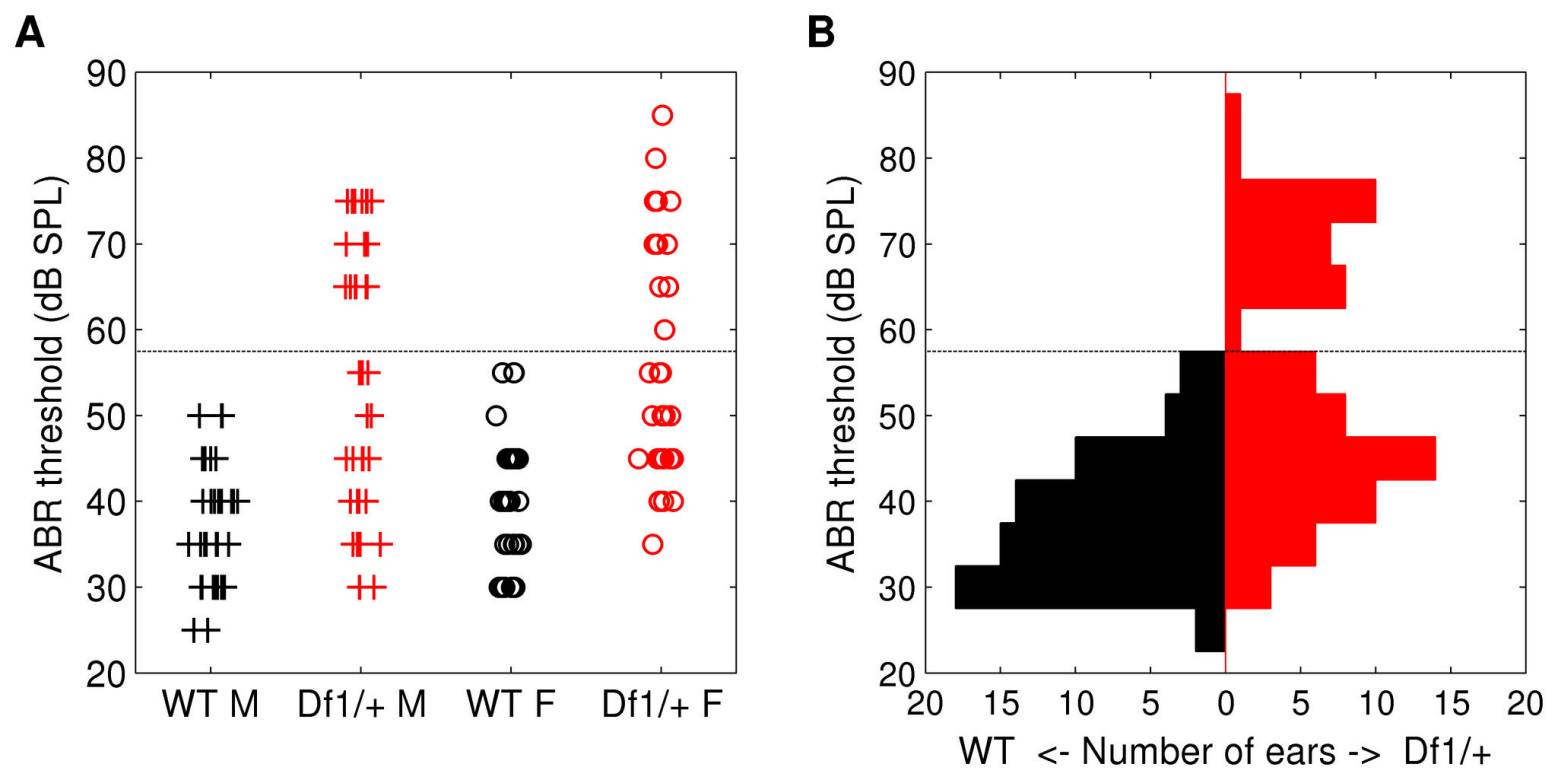
Elevated ABR thresholds, and bimodal distribution of thresholds, in *Df1/+* mice. (A) Click ABR thresholds recorded from individual ears in male and female WT (black) and *Df1/+* (red) mice. Median ABR thresholds differed significantly between *Df1/+* and WT mice of the same gender (Wilcoxon Mann-Whitney test, *Df1/+* versus WT: p=6x10^-6^ males, p=9x10^-7^ females), but not between males and females of the same genotype (Wilcoxon Mann-Whitney test, males versus females: p=0.3 *Df1/+*, p=0.5 WT). (B) Click ABR thresholds pooled across recordings from male and female animals, to illustrate the bimodal appearance of the *Df1/+* ABR threshold distribution. Dashed lines indicate the criterion threshold for a significant click ABR deficit (see text).

Click ABR thresholds were significantly higher on average, and also more variable, in both male and female *Df1/+* mice than in their gender-matched WT littermates (Figure 1A). Median thresholds (and total ranges) were 35 (25-50) and 37.5 (30-55) dB SPL for male and female WT animals, respectively, but 50 (30-75) and 50 (35-85) for male and female *Df1/+* mice from the same litters. Median thresholds therefore differed significantly between recordings from *Df1/+* and WT mice of the same gender (Wilcoxon Mann-Whitney test, *Df1/+* versus WT: p=6x10^-6^ males, p=9x10^-7^ females), but not between males and females of the same genotype (Wilcoxon Mann-Whitney test, males versus females: p=0.3 *Df1/+*, p=0.5 WT). Similar results were obtained when recordings from left or right ears were considered separately.

### ABR threshold distribution in *Df1/*+ mice appears bimodal

Since there were no significant differences in click ABR thresholds recorded from male and female animals of the same genotype, we pooled data from male and female mice to examine genotype differences in the threshold distributions more closely (Figure 1B). The distribution of click ABR thresholds recorded from *Df1/+* mice was significantly different from the distribution recorded from WT mice (Kolmogorov-Smirnov test, p=5x10^-8^), even when the two distributions were normalised to align the medians (Kolmogorov-Smirnov test on median-normalised data, p=0.02). In fact, the *Df1/+* threshold distribution appeared bimodal, suggesting that ABR deficits were perhaps restricted to a subset of *Df1/+* animals.

To be conservative, we defined a click ABR deficit to be present when the ABR threshold exceeded 55 dB SPL (criterion threshold indicated by dashed lines in Figure 1A and B), since 55 dB SPL was the highest threshold observed in recordings from WT mice. By definition, none of the ABR thresholds recorded in WT mice exceeded this criterion; however, 38% of thresholds recorded from the ears of *Df1/+* mice did. Since elevated ABR thresholds could occur either in only one ear or in both ears, the percentage of affected animals could in principle differ from the percentage of affected ears. We investigated this issue with further analysis of ABR data from the subset of animals for which both left and right ear ABR recordings had been obtained.

### ABR deficit in *Df1/*+ mice can be either monaural or binaural

**Figure 2:**
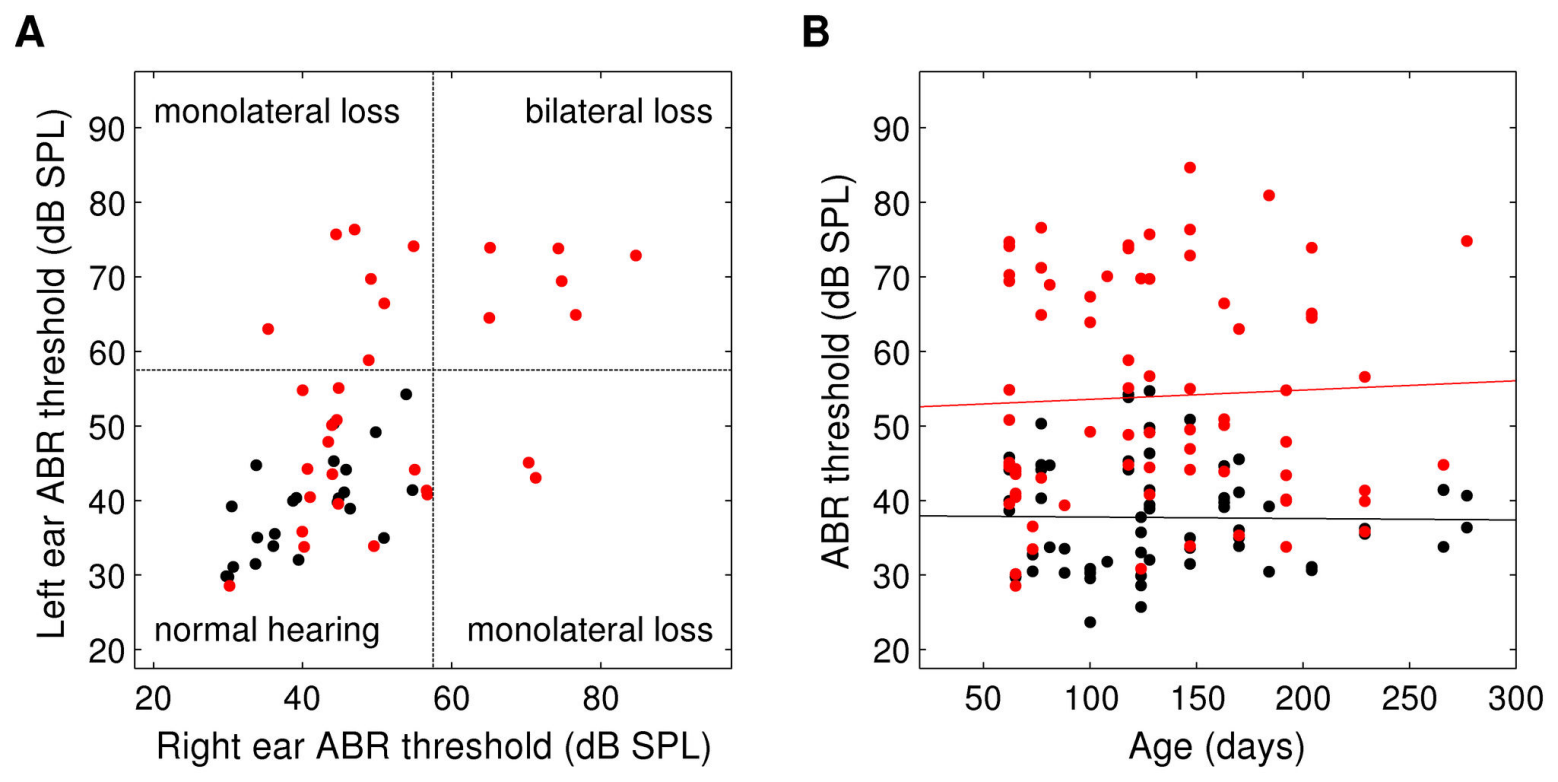
ABR deficit in *Df1/+* mice is often monolateral, and shows no age dependence. (A) Click ABR thresholds recorded in left versus right ears, for WT (black) and *Df1/+* (red) mice in which both ears were tested. Dashed line indicates criterion threshold for a significant click ABR deficit. (B) Click ABR thresholds versus age, for all ABR recordings. Solid lines indicate best-fit regression lines. Slopes were not significantly different from zero for recordings from either WT (black; slope 95% CI [-0.047, 0.072]) or *Df1/+* (red; slope 95% CI [-0.072, 0.030]) animals. To ensure visibility of overlapped data points, zero-mean, 1 dB SPL standard-deviation Gaussian noise was added to threshold data in both A and B for display.

To determine whether click ABR thresholds tended to be similar in the two ears, we compared left and right ABR thresholds for the 31 *Df1/+* and 23 WT animals for which ABR recordings had been collected from both ears (Figure 2A). The correlation between left and right ear ABR thresholds was lower in *Df1/+* than WT mice (Pearson's r=0.49, p=0.006 for *Df1/+* mice; r=0.68, p=0.0004 for WT mice), but not significantly so (Fisher transformation test for difference in correlation coefficients). Thus left and right ear ABR thresholds were correlated in both groups of animals. However, among the *Df1/+* mice, 16 (52%) had no significant click ABR deficit in either ear, 9 (29%) had a significant deficit in one ear, and 6 (19%) had deficits in both ears. Thus, 48% of the *Df1/+* mice had a click ABR deficit in at least one of the two ears, and the deficit was monolateral in 60% of those affected animals. This finding is suggestive of a conductive origin for the hearing loss, because most causes of sensorineural hearing loss would be expected to affect both ears.

### ABR deficit in adult *Df1/*+ mice shows no age dependence

The C57BL/6 background strain from which *Df1/+* mice and their WT littermates are derived is known to have age-related sensorineural hearing loss, especially at high sound frequencies [27,28]. Mutations can accelerate age-related hearing loss, so we wondered if ABR deficits in *Df1/+* mice might worsen with age in adulthood. However, we found no significant dependence of click ABR thresholds on age in adulthood, for either *Df1/+* or WT mice (Figure 2B). Similar results were obtained whether the analysis was performed on all recorded ABR thresholds as shown in Figure 2B, or on thresholds from left ears or right ears separately. These results demonstrate that click ABR deficits in *Df1/+* animals cannot be explained by aging-related effects, suggesting again that these deficits are likely to be primarily conductive in origin. However, since WT C57BL/6 animals would be expected to have age-related hearing loss for high sound frequencies, the negative results for WT animals also indicate that ABR thresholds for a broadband click stimulus are not a sufficiently sensitive assay to evaluate the possibility of high-frequency sensorineural hearing loss in addition to conductive loss (see Discussion).

### Adult *Df1/*+ mice have a high incidence of OM

**Figure 3:**
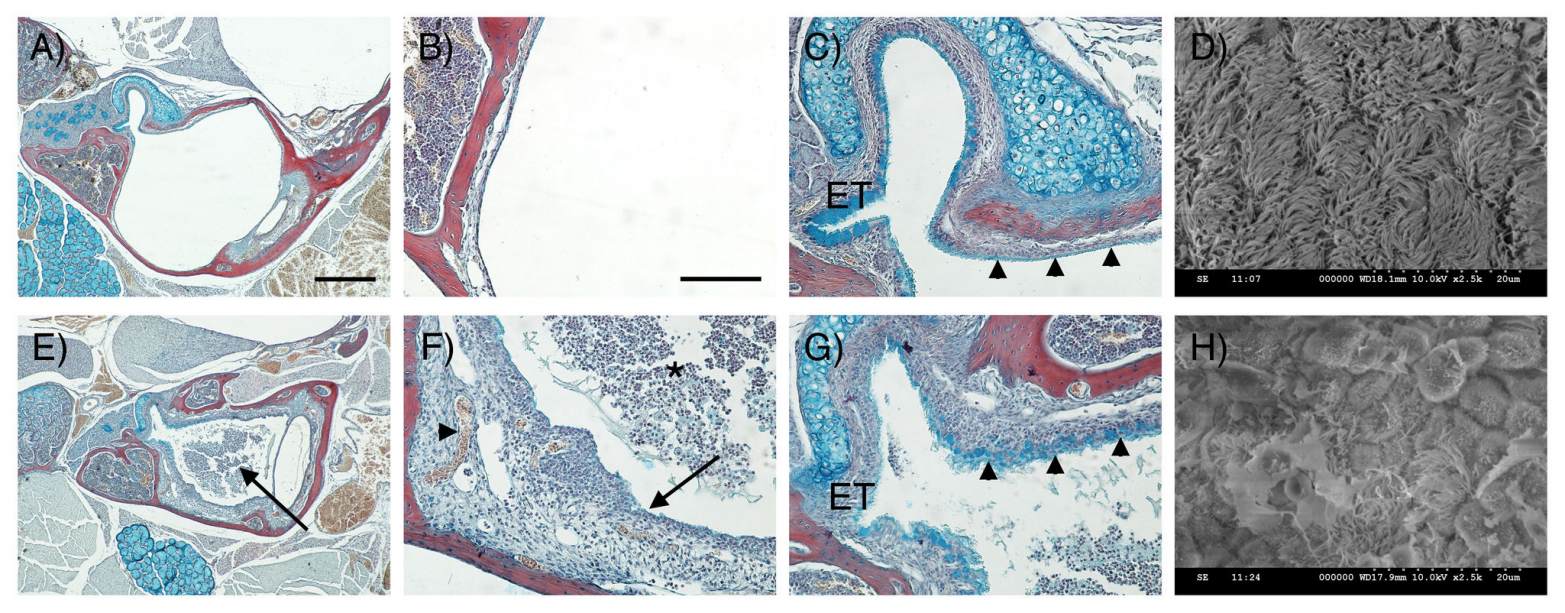
Morphological changes in MEC mucosa and epithelium in *Df1/+* mice. (A-C, E-G) Frontal trichrome-stained sections from 11.5-week-old mice showing the middle ear cavity. (D, H) SEM images of middle ear epithelium. (A-D) WT. (E-H) *Df1/+* with signs of OM. (A) The middle ear cavity is air-filled in the WT. (B) The mucosa is a thin layer lining the auditory bulla. (C) At the entrance of the Eustachian tube (ET) high levels of alcian blue staining are observed indicating mucin production. Further into the middle ear away from the orifice, staining is less distinct in the WT (arrowheads). (D) A thick lawn of cilia is observed overlying the epithelium near the ET orifice. (E) In *Df1/+* mice the middle ear cavity is filled with effusion (arrow). (F) *Df1/+* mice show signs of inflammation such as effusion with infiltrated inflammatory cells (asterix), a thickened mucosa (arrow) and hypervascularisation (arrowhead). (G) In addition, increased alcian blue staining is observed within the middle ear at a distance from the ET indicating increased mucin production in *Df1/+* mice (compare C and G, arrowheads). (H) *Df1/+* mice with OM show reduced numbers of cilia that appear shortened and rarefied. Dorsal is top in A-C, E-G. Scale bar: 500 μm (A, E), 100 μm (B, C, F, G).

To determine whether *Df1/+* mice have middle ear problems, we first examined middle ear anatomy and histology in 9 *Df1/+* mice and their WT littermates. The 9 *Df1/+* mice included 6 animals with confirmed ABR deficits and 3 mice that had not undergone ABR testing; 2 of these 3 mice had a negative Preyer reflex. MicroCT scans revealed no gross abnormalities in middle ear anatomy in the *Df1/+* mice compared to their WT littermates, and there were no defects in the ossicular chain (Figure S2). However, histological analysis demonstrated a high incidence of OME in the *Df1/+* animals (Figure 3E), with frequent signs of inflammation such as effusion, capillary hyperplasia, a thickened tympanic membrane and thickened MEC mucosa (Figure 3F). Affected animals had effusion in one or both ears and the effusion content varied with respect to quantity of infiltrated and inflammatory cells. In some *Df1/+* mice with severe OME, the Eustachian tube (ET) was infiltrated by inflammatory cells. Examination of the mucociliary integrity in *Df1/+* mice with severe OM revealed increased mucus production within the middle ear adjacent to the orifice where the ET enters the MEC, suggesting increased goblet cell density (Figure 3G compared to C). In *Df1/+* mice displaying mild OM, however, this increased mucus secretion was only observed occasionally (data not shown). 

### Morphological changes in the MEC mucosa in *Df1/*+ mice with OM

To further investigate OM-associated changes in the pseudostratified mucociliary epithelium lining the MEC, we turned to scanning electron microscopy (SEM). Comparing the density and distribution of cilia next to the opening of the ET, we observed that WT and *Df1/+* mice without infiltrated cells in the MEC displayed a thick lawn of evenly distributed cilia adjacent to a border region of unciliated epithelium (Figure 3D). In *Df1/+* mice with OME, however, cilia density was reduced and the cilia were rarefied and shortened; in addition, the MEC epithelium was swollen and partly covered in exudate (Figure 3H).

### Elevation of ABR thresholds correlates with the severity of OM in *Df1/*+ mice

To determine whether OM could account for click ABR deficits in Df1/+ mice, we performed histological analysis of the MEC on a set of adult littermates (5 Df1/+ and 1 WT, age 8 weeks) for which click ABRs had been recorded in both ears. The WT mouse and 1 Df1/+ mouse had normal ABR thresholds in both ears; 1 Df1/+ mouse had a slightly elevated ABR threshold in one ear and a normal threshold in the other; 2 Df1/+ mice had a significantly elevated ABR threshold in one ear and a normal or only slightly elevated threshold in the other ear; and 1 Df1/+ mouse had significantly elevated thresholds in both ears. Data from these 6 littermates (12 ears) therefore provided a perfect opportunity to test for a correlation between elevation of click ABR thresholds and signs of OM in Df1/+ animals (Table 1).

**Table 1:** Correlation between severity of OM and click ABR thresholds in ears from six littermates (5 *Df1/+*, 1 WT).

**Signs of OM**		**Click ABR Thresholds**			
**Measure**	**Severity**	**Normal**	**Slightly**	**Elevated**	**Severely**
		**(<50 dB SPL)**	**elevated**	**(60-70 dB SPL)**	**Elevated**
			**(50-55 dB SPL)**		**(>70 dB SPL)**
Effusion	No effusion	3+2*	1	0	0
	Serous effusion	1	1	0	0
	Effusion with <50% infiltrated cells	0	0	2	0
	Effusion with >50% infiltrated cells	0	0	0	2
Mucosa thickening	No thickening (0.077-0.15mm)	2+2*	1	0	0
	Mild thickening (0.151-0.49mm)	2	1	1	0
	Severe thickening (0.491-0.827mm)	0	0	1	2

Degree of effusion with infiltrated cells and thickening of the middle ear mucosa directly correlates with elevation of ABR thresholds in ears from the Df1/+ littermates (Spearman's correlation test: rho=0.88, p=0.0007 for severity of effusion; rho=0.75, p=0.013 for severity of mucosa thickening). Ears from the WT littermate, indicated by the asterix (*) , had normal ABR thresholds and no signs of OM.

Two measures of the severity of OM were used: presence of effusion, and increased thickness of the middle ear mucosa. Analysis was performed blind to genotype and was repeated by multiple operators to ensure reliability of classifications. 

**Figure 4:**
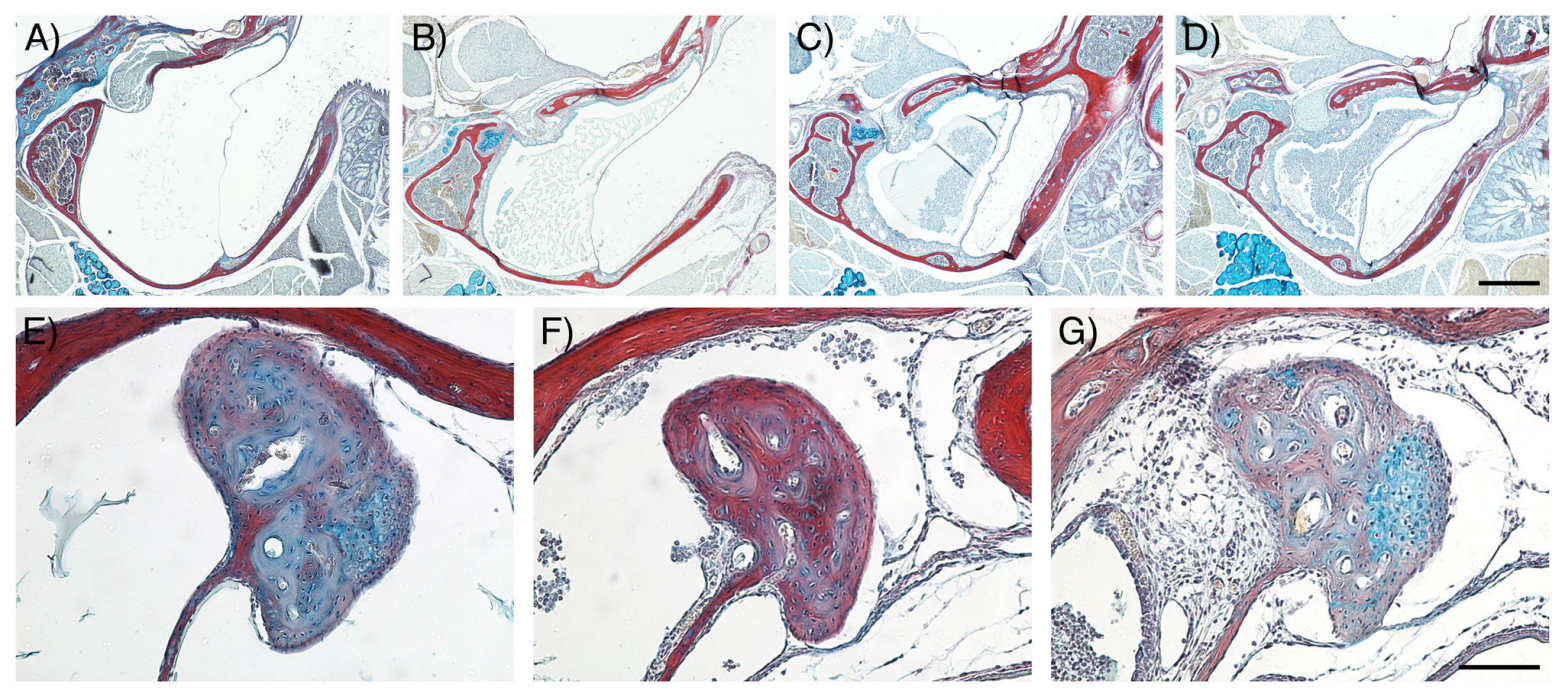
The severity of OM correlates with the degree of hearing loss in *Df1/+* mice (see also Table 1). (A-G) Frontal trichrome-stained sections from adult mice showing the graded severity of effusion in *Df1/+* middle ear cavities (A-D) and thickened mucosa around the head of the malleus (E-G). Least severe conditions are displayed on the left hand side panels with increasing severity towards the right hand side panels. (A) No effusion, (B) serous effusion, (C) effusion with <50% infiltrated cells and (D) effusion with >50% infiltrated cells. (E) No thickening, (F) mild thickening and (G) severe thickening of the mucosa around the head of the malleus. Dorsal is top. Scale bar: 500 μm (A-D), 100 μm (E-G).

As shown in Table 1 and Figure 4, Df1/+ ears for which click ABR thresholds were most elevated (>70 dB SPL) had effusion with more than 50% of infiltrated cells within the MEC (Table 1 and Figure 4D). These mice also displayed a severe thickening of the ME mucosa, which could have produced physical obstruction of movement of the ossicles (Figure 4G). In some cases the epithelial thickness was observed to be up to 23 times higher than in WT littermates. 

Df1/+ ears for which ABR recordings had revealed marginally lower thresholds (60-70 dB SPL) displayed effusion with fewer infiltrated cells and a less severe thickening of the mucosa (Table 1 and Figure 4C), indicating a less advanced inflammation. In these animals there was only limited tissue around the ossicles (Figure 4F).

Df1/+ ears with normal or slightly elevated ABR thresholds (40-45 or 50-55 dB SPL) showed either no effusion (Table 1 and Figure 4A) or a serous effusion only (Table 1 and Figure 4B) with no or mild thickening of the mucosa (Figure 4E or F). Thus both the severity of effusion and the severity of mucosa thickening were significantly correlated with elevation of click ABR thresholds across the Df1/+ ears (Table 1; Spearman's correlation test: rho=0.88, p=0.0007 for severity of effusion; rho=0.75, p=0.013 for severity of mucosa thickening). The two ears from the WT animal (2* in Table 1), for which both ABR thresholds were normal, had no effusion (Figure 4A) and normal ME mucosa (Figure 4E).

In addition to these 6 littermates, we also examined 5 more animals which also underwent hearing tests, and we found the same correlation between hearing loss and OM in those additional animals. No evidence of ossicle erosion was observed, but such erosion might only be present in older mice after repeated bouts of OM.

### Further observations

#### Bacteriology

Bacteriological analysis of middle ear swabs obtained from both ears of 4 *Df1/+* mice revealed, in 4 out of the 8 ears, the presence of commensal bacteria and opportunistic pathogens that do not normally cause infections in a healthy ear. One mouse had scant growth of *Escherichia coli* in both ears; another had moderate growth of *Lactococcus lactis* *spp* *lactis* in one ear; a third was found to have scant growth of *Pantoea* *spp* in one ear. No fungi or yeast were isolated from any of the samples. These results suggest that OM in *Df1/+* mice is unlikely to be caused by unusual susceptibility to a specific pathogen; rather, any bacterial infection of the middle ear in *Df1/+* mice is probably a secondary opportunistic process.

#### Hair cell density in the organ of Corti

To address the possibility that elevated ABR thresholds in *Df1/+* mice might arise from sensorineural as well as conductive hearing loss, we examined the sensory epithelium of the inner ear in 6 *Df1/+* and 5 WT mice. There was no evidence for significant loss of hair cells sufficient to account for the elevated ABR thresholds in *Df1/+* relative to WT mice. Moreover, the density of hair cells in the cochlea appeared normal in both ears of *Df1/+* animals with monolateral hearing loss (Figure S3). On the basis of our analysis, we cannot entirely rule out the possibility of subtle inner ear abnormalities in *Df1/+* mice, especially given that severe OM can sometimes affect the inner ear. However, we can conclude that the observed click ABR deficit in *Df1/+* mice does not arise from hair cell loss.

## Discussion

Here we have shown that *Df1/+* mice are susceptible to conductive hearing loss and otitis media, which are also common consequences of the human 22q11.2 deletion that the mice were genetically engineered to model. Mouse models of 22q11DS such as the *Df1/+* mouse have attracted great interest not only as a tool for investigating the origins of various defects and disabilities associated with this relatively common chromosomal disorder, but also as a means of gaining insight into the pathogenesis of schizophrenia, for which 22q11DS is one of the most significant known risk factors. In the context of this previous research, the present study makes three distinct contributions.

First, the results suggest a resolution to a discrepancy in the literature between previous studies reporting normal hearing in the *Df1/+* and *Df(16*)*A/+* mouse models of 22q11DS [23-25] and studies documenting a high incidence of middle ear disease in mice heterozygous for the gene *Tbx1* [26], which is included in the *Df1/+* and *Df(16*)*A/+* deletion regions. The previous evidence for normal hearing in mouse models of 22q11DS came from supplementary controls in studies of prepulse inhibition (PPI) of acoustic startle. In one study [23], thresholds for acoustic startle were observed to be slightly lower, and startle amplitudes slightly higher, in *Df1/+* mice than in their WT littermates; in another study [25], similar results were reported for *Df(16*)*A/+* mice, which have a larger deletion region than *Df1/+* mice. Both studies therefore concluded that there was no evidence for reduction in hearing sensitivity in these mice. However, under some circumstances, mice with partial hearing loss can show reduced startle thresholds and elevated startle amplitudes in acoustic startle testing [29], perhaps because central auditory adaptation to the reduction in peripheral input leads to hyperacusis for loud sounds. Better evidence for normal hearing in *Df1/+* mice was provided in [24] based on frequency-specific distortion-product otoacoustic emission (DPOAE) testing, which is generally an excellent means of detecting peripheral auditory abnormalities including middle ear problems. However, the DPOAE testing in that study was performed on only 6 *Df1/+* mice, and apparently only on one ear in each animal. Given the intermittent nature of the ABR deficit and OM observed in the present study (48% of *Df1/+* animals, but only 38% of *Df1/+* ears tested), it is possible that this sample size was too small. We therefore suggest that previous evidence for normal hearing in *Df1/+* and *Df(16*)*A/+* mice was inconclusive, and that all mouse models of 22q11DS involving deletion of *Tbx1* may have a high incidence of conductive hearing loss.

The second contribution of the present work is to show that previous reports of abnormal auditory sensorimotor gating in mouse models of 22q11DS need to be re-examined to determine whether abnormalities in auditory processing alone might account for the results. Impaired auditory sensorimotor gating, quantified as a reduction in PPI of acoustic startle, is considered an important endophenotype for risk of schizophrenia. Reduced PPI of acoustic startle has been reported both in human 22q11DS patients [30] and in mouse models of 22q11DS [23-25]. However, as discussed above, the high incidence of conductive hearing loss and otitis media in *Df1/+* mice could itself lead to differences between *Df1/+* and WT animals in PPI of acoustic startle. Moreover, although we found no abnormalities in hair-cell density that could account for the observed elevation of click ABR thresholds in *Df1/+* mice, it is possible that *Df1/+* mice have more subtle sensorineural hearing deficits, especially given that *Tbx1* is involved in inner ear development [31]. The possibility of such abnormalities could be explored further in the future with tone-evoked ABR measurements or distortion-product otoacoustic emission (DPOAE) testing, which are more sensitive assays for frequency-specific hearing loss than click-evoked ABR measurements.

The final contribution of this work is to introduce the *Df1/+* mouse as a powerful model system for investigating the pathogenesis of OM in 22q11DS and also more generally. Hearing loss is prevalent among human 22q11DS patients [32], and is almost always related to middle ear infection. In one study, chronic or recurrent otitis media was reported in 52% of 22q11DS patients [18]; in another 88% had otitis media, 53% had conductive hearing loss, and 39% required surgical implantation of ventilation tubes to drain the middle ear [17]. In these patients the causes of the OM have been proposed to be multifactorial involving immune deficiency, palatal abnormalities and Eustachian tube dysfunction [18]. The high incidence of conductive hearing loss and otitis media in *Df1/+* mice indicates that these animals can be used to tease apart the causes of frequent OM in 22q11DS patients. Moreover, the prevalence of monolateral ABR deficits and OM in *Df1/+* mice creates opportunities for within-animal controls, making the animals a potentially powerful tool for testing hypotheses about the causes of OM. Studies of the mechanism by which genetic predispositions cause OM have been performed in other mouse models, such as the heterozygous *Fbxo11* mouse and *Eya4* knockout mouse. *Fbxo11* is expressed in the lining of the middle ear cavity and has been proposed to affect epithelial inflammatory events in the ear [33]. In contrast, in the *Eya4* mouse the morphology of the Eustachian tube and angle of connection to the middle ear have been shown to be defective, potentially causing ear clearance problems [34]. *Tbx1* has been shown to be expressed in the endoderm of the developing pharyngeal arches and *Tbx1* null mice have a hypoplastic pharynx [35,36]. As the Eustachian tube arises from the endodermally derived first pharyngeal pouch it is therefore tempting to speculate that a subtle early defect in patterning of the endoderm might be responsible for the high incidence of OM in *Df1/+* and *Tbx1* heterozygous mice. Our ongoing efforts to pinpoint the causes of otitis media in *Df1/+* mice are therefore focusing on the possibility of abnormalities in the morphology of the Eustachian tube and/or the other endodermally derived tissues of the middle ear [37]. 

In conclusion: *Df1/+* mice, like human 22q11DS patients, are susceptible to otitis media and conductive hearing loss, which affect nearly half the animals but often in only one ear. The findings suggest that abnormal auditory sensorimotor gating previously reported in mouse models of 22q11DS could arise from abnormalities in auditory processing. More broadly, the results indicate that *Df1/+* mice are an important model system for investigating the causes of OM in both 22q11DS patients and the many children worldwide who suffer from chronic middle ear infections. 

## Materials and Methods

### Ethics statement

All *in vivo* experiments were conducted in accordance with the United Kingdom's Animal (Scientific Procedures) Act of 1986, under a project licence approved by the UK Home Office.

### Animals

The *Df1/+* mouse line had been maintained on a C57BL/6 background for a minimum of 10 generations prior to the analyses. The *Df1* deletion itself was engineered on a 129S5 SvEvBrd genetic background [21]. 

### Hearing tests

#### Auditory brainstem response measurement

ABR testing was performed in a sound isolation booth (Industrial Acoustics Company, Inc.). Mice were anaesthetised with ketamine and medetomidine. Body temperature was maintained at 37-38°C using a homeothermic blanket (Harvard Apparatus). Subdermal needle electrodes (Rochester Medical) were inserted under the skin with positive electrode at the vertex, negative electrode near the ear being tested, and ground electrode near the opposite ear (which was blocked with a sound-attenuating earplug). For most animals, ABR recordings were obtained from both the left and right ears in turn, with an earplug in the ear opposite to that under test (to ensure monaural stimulus presentation).

Auditory stimuli were presented via a free-field speaker (FF1) from Tucker-Davis Technologies (TDT). Speaker output was calibrated before each set of experiments using a Bruel & Kjaer ¼ inch microphone (4939), placed at the location of the ear to be tested. Stimulus generation and data acquisition were accomplished using hardware from TDT (RX6 and RX5 signal processors, RA4LI and RA16SD signal amplifiers, PA5 attenuator, and SA1 speaker amplifier), a custom low-pass filter designed to remove attenuation switching transients, and software from TDT (Brainware) and Mathworks (Matlab). 

Stimuli were 50 μs monophasic clicks ranging in sound level from 0 to 90 dB SPL, presented at a rate of 20 clicks/sec. ABR recordings typically included 500 repeats of click stimuli presented over a 20-80 dB SPL range at 20 dB intensity increments, followed by 1000 repeats of click stimuli presented over a smaller intensity range at 5 dB intensity increments. The threshold was defined to be the lowest click intensity evoking a clear and characteristic deflection of the ABR wave that was at least as large as the time-dependent standard error in the mean wave at that sound intensity.

#### Preyer reflex assessment

A few of the mice used for studies of middle ear anatomy and histology did not undergo ABR measurement due to time constraints, and instead were tested for a Preyer reflex. The Preyer reflex is a flick of the pinnae evoked by a transient loud sound. To present such sounds, we used a custom-built click box (MRC Institute of Hearing Research, Nottingham, UK) emitting a brief 18.5 kHz tone burst with intensity 95-105 dB SPL at the distances typically used for testing. Since the Preyer reflex is somewhat unreliable even in animals with normal hearing, we judged the Preyer reflex to be negative only if an animal showed no Preyer reflex across several presentations of the sound.

### Middle ear analyses

#### Histology

Mouse heads were fixed in 4% paraformaldehyde (PFA) overnight at 4°C and then decalcified in EDTA Solution (67.5% EDTA, 7.5% PBS and 25% PFA (4%)). The tissue was then dehydrated through a methanol series and isopropanol and cleared in tetrahydronaphthalene before embedding in paraffin wax. The 9 μm frontal sections were mounted on Superfrost Plus Slides, dewaxed in Histoclear, rehydrated through IMS, stained with 1% Alcian blue in 3% acetic acid pH2.5, Ehrlich’s haematoxylin, and 0.5% Sirius Red in saturated picric acid, and then mounted in DPX. Slides were imaged on a Nikon Digital Sight Camera. Measurement of mucosa thickness was performed using Image J software.

#### Scanning electron microscopy

The temporal bones of adult mice were dissected and the middle ear mucosa revealed by removing the outer ear, eardrum, tympanic ring and the malleus and incus. These were then fixed in 2.5% gluteraldehyde in 0.15M cacodylate buffer (pH7.2) overnight at 4°C, and washed and postfixed in 1% osmium tetroxide. Next specimens were dehydrated through an ethanol series and dried using a Polaron E3000 critical point dryer. After mounting and coating with gold (Emitech K550X sputter coater), the surface of the mucosa was examined and images recorded using a Hitachi S-3500N scanning electron microscope (SEM) operated at 10kV in high vacuum mode.

#### MicroCT reconstruction

Micro computerized tomography (microCT) was used for the three-dimensional analysis of *Df1/+* and WT middle ear morphology.

### Inner ear analysis

Cochleae were fixed in 4% PFA in PBS for 2 hrs, then decalcified in 4% EDTA in PBS at pH7.4 for 48 hrs at 4°C. The organ of Corti was extracted in half-turn segments, permeabilised in 0.5% Triton X-100 for 15 min, and incubated with fluorescently conjugated phalloidin at 1 μg/ml for 2 hrs. Phalloidin labels filamentous actin and therefore delineates both hair cell stereocilia and intercellular junctions at the luminal surface of the organ of Corti. Segments were mounted on slides using an anti-photobleaching agent (Vectashield) that also contained DAPI (VectaLabs) to label cell nuclei. Slides were then examined and images taken with a confocal microscope (Zeiss) and viewed for analysis through LSM browser (Zeiss).

## Supporting Information

Figure S1 **Example click ABR waveforms from a WT mouse (**A**) and a *Df1/+* littermate (B), both male and 29 weeks old.** Left plots show ABR waveforms evoked by clicks at 20, 40, 60 and 80 dB SPL, averaged over 500 trials for each stimulus. Right plots show ABR waveforms evoked by clicks presented over a smaller intensity range and at finer intensity resolution, averaged over 1000 trials per stimulus. Grey shading around black lines indicates standard error of the mean across trials. Threshold was judged to be 30 dB SPL for the WT mouse (A), and 65 dB SPL for the *Df1/+* animal (B).
(TIF)

Figure S2 **Middle ear structure in adult WT and *Df1/+* mice.** MicroCT reconstruction of the bony auditory bulla surrounding the middle ear cavity, with ossicles shown in pseudocolor. No differences in morphology of the middle ear and the ossicular chain were observed between (A) WT and (B) *Df1/+* mice. Scale bar: 1mm.
(TIF)

Figure S3 **Whole-mount organ of Corti segments taken from the basal turns of the left (**A**) and right (**B**) cochleae in a male *Df1/+* mouse (age 24 weeks) with pronounced monolateral hearing loss.** ABR thresholds measured *in* *vivo* were 65 dB SPL for the left ear and 35 dB SPL for the right ear. In both ears, the sensory epithelium appears relatively normal for an animal of this age, with only the occasional hair cell missing. Red, phalloidin stain (highlighting filamentous actin in hair cells); blue, DAPI (highlighting cell nuclei). Scale bars: 20 μm.
(TIF)
