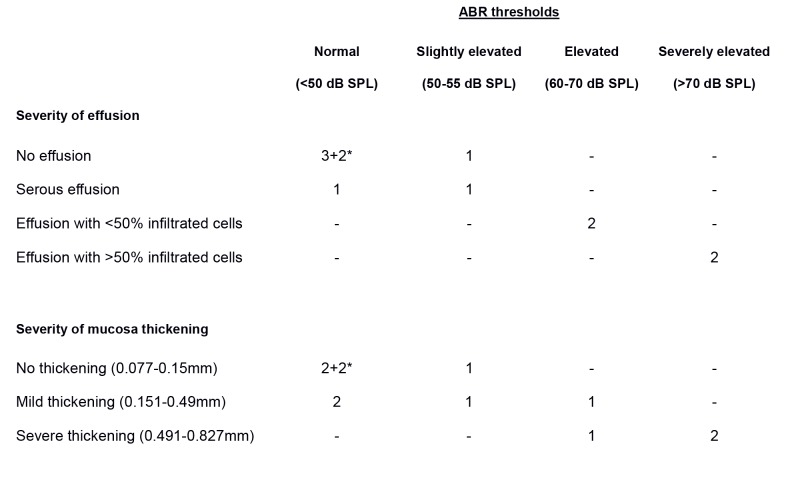# Correction: Hearing Loss in a Mouse Model of 22q11.2 Deletion Syndrome

**DOI:** 10.1371/annotation/f10a686b-b6e2-4630-8c6f-7a91191ae732

**Published:** 2014-01-02

**Authors:** Jennifer C. Fuchs, Fhatarah A. Zinnamon, Ruth R. Taylor, Sarah Ivins, Peter J. Scambler, Andrew Forge, Abigail S. Tucker, Jennifer F. Linden

There was a formatting error in Table 1, which has been corrected for improved readability. Please see the corrected Table 1 here: 

**Figure:**