# Supplementary figures and images for: Hearing Loss in a Mouse Model of 22q11.2 Deletion Syndrome
Source: PLoS One. 2013 Nov 14;8(11):e80104. doi: 10.1371/journal.pone.0080104 (PMC3828191; doi:10.1371/journal.pone.0080104)

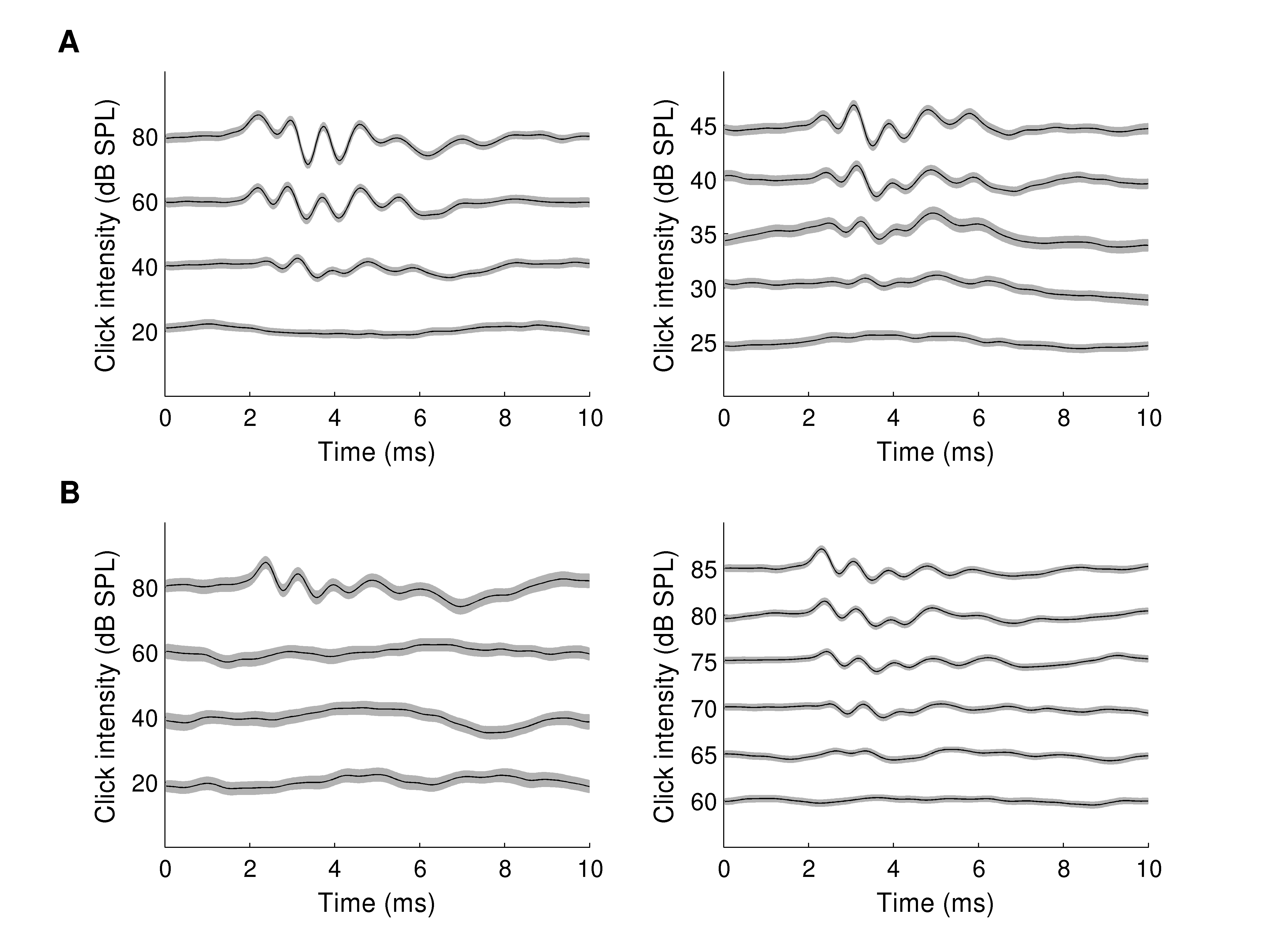

Supplement: Figure S1 — Example click ABR waveforms from a WT mouse (A) and a Df1/+ littermate (B), both male and 29 weeks old. Left plots show ABR waveforms evoked by clicks at 20, 40, 60 and 80 dB SPL, averaged over 500 trials for each stimulus. Right plots show ABR waveforms evoked by clicks presented over a smaller intensity range and at finer intensity resolution, averaged over 1000 trials per stimulus. Grey shading around black lines indicates standard error of the mean across trials. Threshold was judged to be 30 dB SPL for the WT mouse (A), and 65 dB SPL for the Df1/+ animal (B). (TIF) [file pone.0080104.s001.tif]

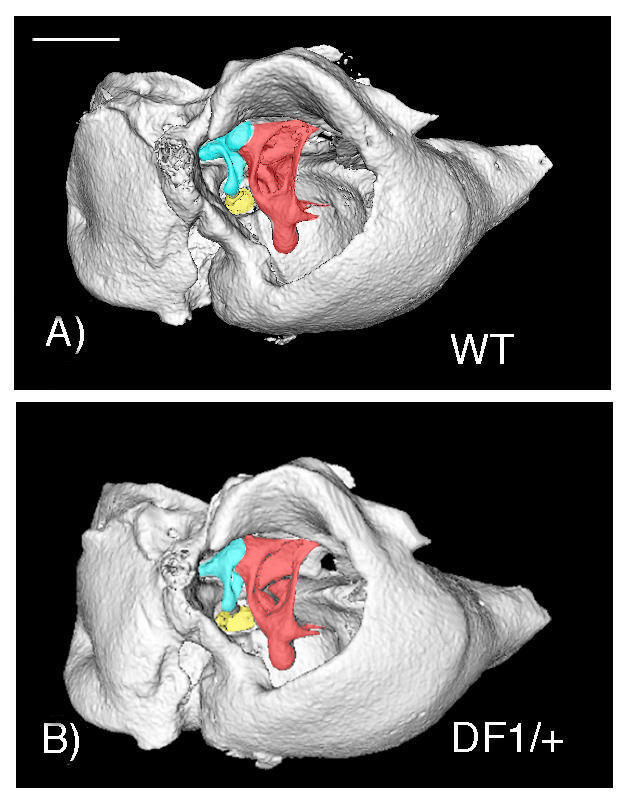

Supplement: Figure S2 — Middle ear structure in adult WT and Df1/+ mice. MicroCT reconstruction of the bony auditory bulla surrounding the middle ear cavity, with ossicles shown in pseudocolor. No differences in morphology of the middle ear and the ossicular chain were observed between (A) WT and (B) Df1/+ mice. Scale bar: 1mm. (TIF) [file pone.0080104.s002.tif]

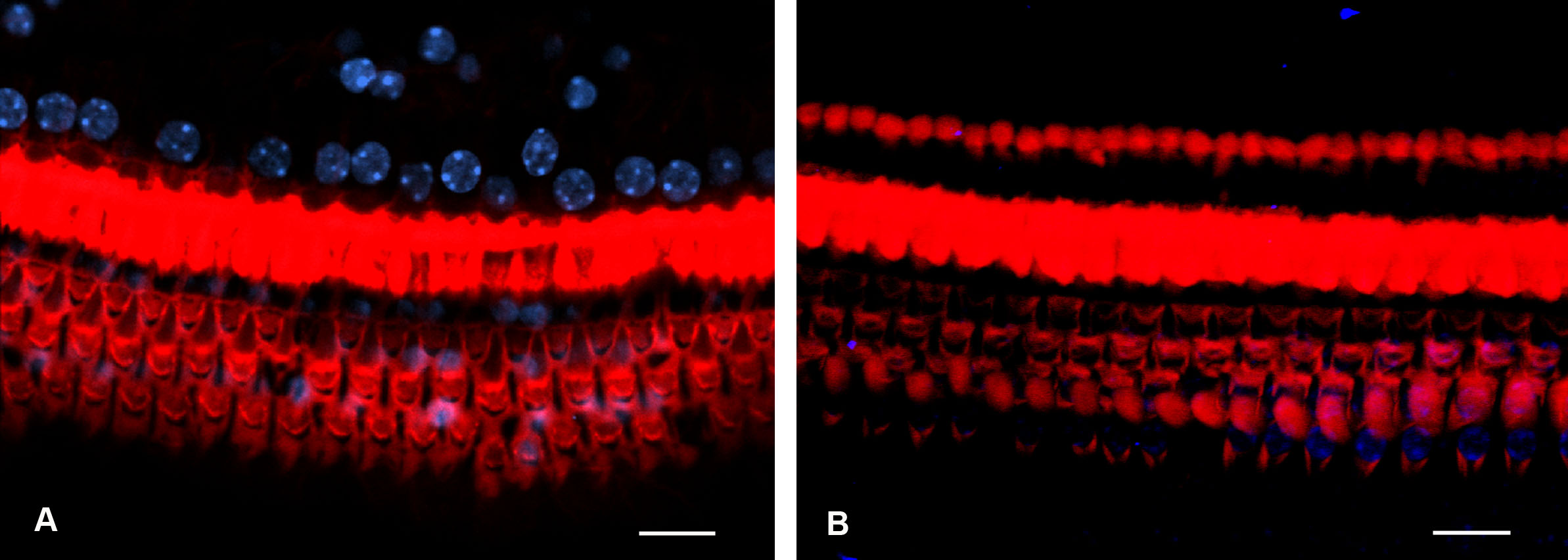

Supplement: Figure S3 — Whole-mount organ of Corti segments taken from the basal turns of the left (A) and right (B) cochleae in a male Df1/+ mouse (age 24 weeks) with pronounced monolateral hearing loss. ABR thresholds measured in vivo were 65 dB SPL for the left ear and 35 dB SPL for the right ear. In both ears, the sensory epithelium appears relatively normal for an animal of this age, with only the occasional hair cell missing. Red, phalloidin stain (highlighting filamentous actin in hair cells); blue, DAPI (highlighting cell nuclei). Scale bars: 20 μm. (TIF) [file pone.0080104.s003.tif]
